# Long Non-coding RNA MIR570MG Causes Regorafenib Resistance in Colon Cancer by Repressing miR-145/SMAD3 Signaling

**DOI:** 10.3389/fonc.2020.00291

**Published:** 2020-03-05

**Authors:** Fang Wei, Mofei Wang, Zhen Li, Yong Wang, Yong Zhou

**Affiliations:** Department of General Surgery, The Fourth Affiliated Hospital of China Medical University, Shenyang, China

**Keywords:** LncRNA MIR570MG, miR-145, SMAD3, TGFβ, metastatic colon cancer, regorafenib, resistance

## Abstract

An increasing number of studies have shown that long non-coding RNA (lncRNA) dysregulation plays a fundamental role in the development of various cancers, including colon cancer. Nonetheless, the mechanisms of lncRNA in regorafenib-resistance remain unclear. Our research revealed the lncRNA MIR570MG increased in regorafenib-resistant colon cancer cells compared to the regorafenib-sensitive cells. Furthermore, MIR570MG sponged miR-145, which declined in regorafenib-resistant colon cancer cell lines. More importantly, overexpression of miR-145 hampered cell proliferation and retrieved colon cancer regorafenib-sensitivity, contrary to the function of MIR570MG. Dual-luciferase reporter assay confirmed that miR-145 bound to 3′-UTR of SMAD3, a transcriptional modulator activated by TGFβ, resulting in blockage of TGFβ /SMAD3-mediated cell growth and cycle progression. Besides, ectopic expression of miR-145 inhibitor in the parental cells endowed resistance to regorafenib. Inversely, knockdown of MIR570MG impoverished resistance against regorafenib. Additionally, overexpression of MIR570MG conquered the suppression of tumor growth by miR-146 and rehabilitated the resistance to regorafenib in HCT116R human colon cancer mouse models. In summary, our findings suggested that MIR570MG promoted regorafenib resistance via releasing SMAD3 from miR-145, leading to activation of SMAD3-mediated signaling pathways.

## Introduction

Despite the achievement in multimodality therapy combining chemo-, radio- and targeted therapy over the last decade, the mortality and the recurrence rates of metastatic colorectal cancer are inadequate. Novel agents targeting epidermal growth factor receptor, insulin-like growth factor receptor, transforming growth factor-beta receptor and other growth factors were developed to treat advanced/refractory colorectal cancer. Recently, the United States Food and Drug Administrate has approved regorafenib, an oral multi-kinases inhibitor, for treating unresectable or metastatic colon cancer following first- and second-line therapy (1). regorafenib targets RAF, angiogenic, stromal and receptor tyrosine kinases. Nonetheless, the intrinsic and acquired resistance to regorafenib restrains its application further. So far, the exact mechanisms underlying resistance to regorafenib remain mostly unknown.

While the minority of genome encodes proteins, most of the genome comprises non-coding sequences. Most of the non-coding RNA is transcribed to RNA, including long non-coding RNA (lncRNA) with lengths exceeding 200 nucleotides. Increasing numbers of studies have identified that lncRNA, microRNA (miRNA), and protein-coding message RNA (mRNA) compose complex RNA networks by competitive association. Abnormal regulation or aberrant expression of various lncRNA plays a role in the initiation, progression, and relapse of colorectal cancer (2, 3).

At present, we found lncRNA MIR570MG upregulated in regorafenib-resistant cells, antagonizing apoptosis, loss of colony formation, and cell cycle arrest by regorafenib. Conversely, miR-145, one of the sponges of MIR570MG, deregulated in regorafenib-resistant cells. Moreover, we identified miR-145 targeted *SMAD3*. We hypothesized that MIR570MG: miR-145: *SMAD3* association played a pivotal role in acquired resistance to regorafenib.

## Materials and Methods

### Cell Lines and Chemicals

RPMI-1640 and fetal bovine serum (FBS) were purchased from Thermo Fisher Scientific Inc. (Waltham, MA, USA). The human colon cancer cell lines SW480 (catalog number: CCL228) and HCT116 (catalog number: CCL247) were obtained from American Type Culture Collection (Manassas, VA, USA). Cells were grown in RPMI-1640, supplemented with 10% FBS. All cell lines were maintained in a humidified incubator with 5% of CO_2_ and 95% air at 37°C.

Regorafenib (BAY 73-4506) Catalog No. S1178 was purchased from Selleck Chemicals (Houston, TX, USA). Dimethyl sulfoxide (DMSO) and 3-(4,5-dimethylthiazol-2-yl)-2,5-diphenyltetrazolium bromide (MTT) were obtained from Merck & Co. (Kenilworth, NJ, USA). siRNA negative control and siRNA targeting MIR570MG was synthesized in Origene Technologies, Inc. Rockville, MD, USA).

### Construction of Expression Plasmids and Stable Cell Lines

According to the manufacturer's protocols (OriGene Technologies, Inc. Rockville, MD, USA), lncRNA MIR570MG was amplified and inserted into expression vector pLenti-C-mGFP. miR-145 was cloned and inserted into expression vector pCMV-MIR-GFP. HCT116 cells were transfected with pLenti-C-mGFP-MIR570MG followed the standard procedures. Subsequently, cells expressed GFP-MIR570MG stably were selected by a fluorescence microscopy (Olympus IX70, Shinjuku, Tokyo, Japan). The stable sublines were transfected with pCMV-MIR-GFP-miR-145, followed by a selection with Neomycin.

### Establishment of Acquired Regorafenib Resistant Cells

Both SW480 and HCT116 were exposed to stepwise increasing regorafenib for over 12 months. The survival cells were subsequently passaged and grew under the same conditions for more than 20 passages with 0.02 μM regorafenib. The regorafenib-adapted cells named SW480R and HCT116R.

### MTT Assay

Cells were seeded on a 96-well plate and allowed to attach overnight. Different concentrations of regorafenib (range, 0.01–20 μM) or DMSO were used to treat cells. Cell viability was determined with MTT. The half-maximum inhibitory concentration (IC_50_) values were calculated by interpolation from the dose-response curves. Results represented the median of three independent experiments, each performed in triplicate.

### Dual-Luciferase Assay

Wild-type (MI) or MIR570MG mutant (MI Mut), together with miR-145, were transfected into SW480 cells. Furthermore, wild-type (wt) or mutant (Mut) of the miR-145, along with 3′-untranslated region (3′-UTR) of *SMAD3*, were transfected into SW480R cells. Twenty-four hours later, cells were lysed, and the relative light unit was detected by dual luciferase assay followed the manufacturer's protocol (Promega Corporation, Madison, WI, USA).

### Western Blot

Lysis buffer (Thermo Fisher Scientific, Inc., Waltham, MA, USA) was used to extracted protein from cell precipitation. The concentration was measured by a Pierce BCA Protein Assay Kit. A total of 30 μg protein was resolved on a 10% gel using SDS-PAGE. The lysis buffer, BCA Protein Assay Kit, and gel were obtained from Thermo Fisher Scientific, Inc. (Waltham, MA, USA). Subsequently, proteins were transferred to a PVDF membrane (Millipore, Burlington, MA, USA). The membranes were blocked in 5% skimmed milk in TBS-Tween for 1 h at room temperature and incubated with the following primary antibodies: mouse monoclonal antibodies Smad3 (sc-101154), XIAP (sc-55551), cyclin D1 (sc-8396) and Cdk4 (sc-23896) at 4°C overnight, after which time the membranes were incubated with mouse IgG kappa binding protein (m-IgGκ BP) conjugated to Horseradish Peroxidase (sc-516102). GAPDH (sc-47724) served as the loading control. All antibodies were purchased from Santa Cruz Biotechnology, Inc. (Dallas, TX, USA). The dilution of primary antibodies was 1:1,000 and the dilution of the secondary antibody were 1:2,000. Signals were collected using enhanced chemiluminescence (Pierce; Thermo Fisher Scientific, Inc.).

### Quantitative Reverse Transcript-Polymerase Chain Reaction

Total RNA was extracted using a RNeasy Mini Kit (QIAGEN, Hilden, Germany) according to the manufacturer's protocol. Reverse transcription of RNA to cDNA was performed using a QuantiTect Whole Transcriptome Kit (QIAGEN, Hilden, Germany). PCR amplification was performed with the LightCycler® 480 Block system (Roche, Basel, Switzerland) followed the instrument's protocol. GAPDH served as the housekeeping gene to normalize the expression of lncRNA and mRNAs, while U6 served as the reference gene to normalize miR-145 expression. Gene expression levels were calculated relative to the expression of the housekeeping genes with the ΔΔCq method (4). The primer sequences were as follows: MIR570MG, Forward, 5′-TTGTGCTCATGTGATGGGGG3′, Reverse, 5′-GTGGTATAATGCGCAGAAGGTAAT-3′. *miR-145*, Forward, 5′-GTCCAGTTTTCCCAGGA-3′, Reverse, 5′-GAACATGTCTGCGTATCTC-3′; *SMAD3*, Forward, 5′-TGAGGCTGTCTACCAGTTGACC-3′, Reverse, 5′-GTGAGGACCTTGTCAAGCCACT-3′; *XIAP*, Forward, 5′-TGGCAGATTATGAAGCACGGATC-3′, Reverse, 5′-AGTTAGCCCTCCTCCACAGTGA-3′; *CCND1*, Forward, 5′-TCTACACCGACAACTCCATCCG-3′, Reverse,5′-TCTGGCATTTTGGAGAGGAAGTG-3′; *CDK4*, Forward, 5′-CCATCAGCACAGTTCGTGAGGT-3′, Reverse, 5′-TCAGTTCGGGATGTGGCACAGA-3′; *GAPDH*, Forward, 5′-GTCTCCTCTGACTTCAACAGCG-3′, Reverse, 5′-ACCACCCTGTTGCTGTAGCCAA-3′; *U6*, Forward, 5′-CCGTATGACCTCCTTCCACAGA-3′, Reverse, 5′-TCTGTCCACCTCTGAAACCAGG-3′.

### Cell Cycle Analysis

Cells were aliquoted and placed into 60-mm petri dishes, followed by the previous description (5). The BD CellQuest Pro™ Software (version 5.1) was provided by the Becton, Dickinson and Company Corporate (Franklin Lakes, NJ, USA). Cell cycle analysis was performed with ModFitLT for Windows version 5.0.

### Apoptosis Assay

Cells expressing lncRNA, miRNA, or the combination were exposed to regorafenib. Twenty-four hours later, the JC-1 assay was conducted, followed by flow cytometry. The details were described previously (6). JC-1 dye obtained from Thermo Fisher Scientific (Waltham, MA, USA).

### LncRNA Array

Three clones of SW480R were used for PrimePCR lncRNA array (QIAGEN, Hilden, Germany), and the data were analyzed with the QIAGEN GeneGlobe Data Analysis Center (https://geneglobe.qiagen.com/us/analyze/) followed the manufacture's protocol. Data gained from at least three independent experiments. RNAs (fold change >1.0 and *P* < 0.05) were considered expressed differentially between two groups.

### Prediction of Putative LncRNA, miRNA, and mRNA Association

Interactions between lncRNA and miRNA, as well as the miRNA-mRNA, were predicted with starBase version 2.0 (http://starbase.sysu.edu.cn/index.php) and MicroRNA Target Prediction Database (miRDB, http://www.mirdb.org/) followed the previous instrument (7, 8).

### Generation of Human Colon Cancer Xenografts

All studies were approved by the Medical Ethics Committee of the Fourth Affiliated Hospital of China Medical University and conducted according to the guideline of the Experimental Animal Center of the Fourth Affiliated Hospital of China Medical University. Four to five weeks old BALB/c nude female mice were obtained from Beijing Laboratory Animal Research Center (Beijing, China). Mice were housed in specific-pathogen-free conditions. 2 × 10^6^ HCT116R cells expressing control, miR-145 or miR-145 along with MIR570MG stably was injected in mice subcutaneously. Mice carried approximately 100 mm^3^ tumors were divided into three groups randomly (six per group) post-injection. Mice received daily regorafenib 30 mg/kg body weights by gavage for 4 weeks according to the previous studies (9, 10). Tumor volumes were measured every 4 days with a digital caliper according to the formula V = 0.5 × (W^2^ × L), where V defined as volume, W defined as minor axis and L defined as the major axis of the measurement. Twenty-eight days later, mice were euthanasia with carbon dioxide inhalation. Tumors were resected, weighed and photographed. Besides, the expression of interesting lncRNA, miRNA, and mRNA were accessed by immunoblot and qRT-PCR, respectively.

### Statistical Analysis

The results present as the mean ± standard deviation of three independent experiments. Comparisons of various groups were carried out using two-way ANOVA. Dunnett's multiple comparisons test was performed to compare the mean of each group with the mean of the control group. *P* < 0.05 was considered to indicate a statistically significant difference.

## Results

### Chronic Regorafenib Exposure to Generate Regorafenib-Resistant Cells

**Figure 1:**
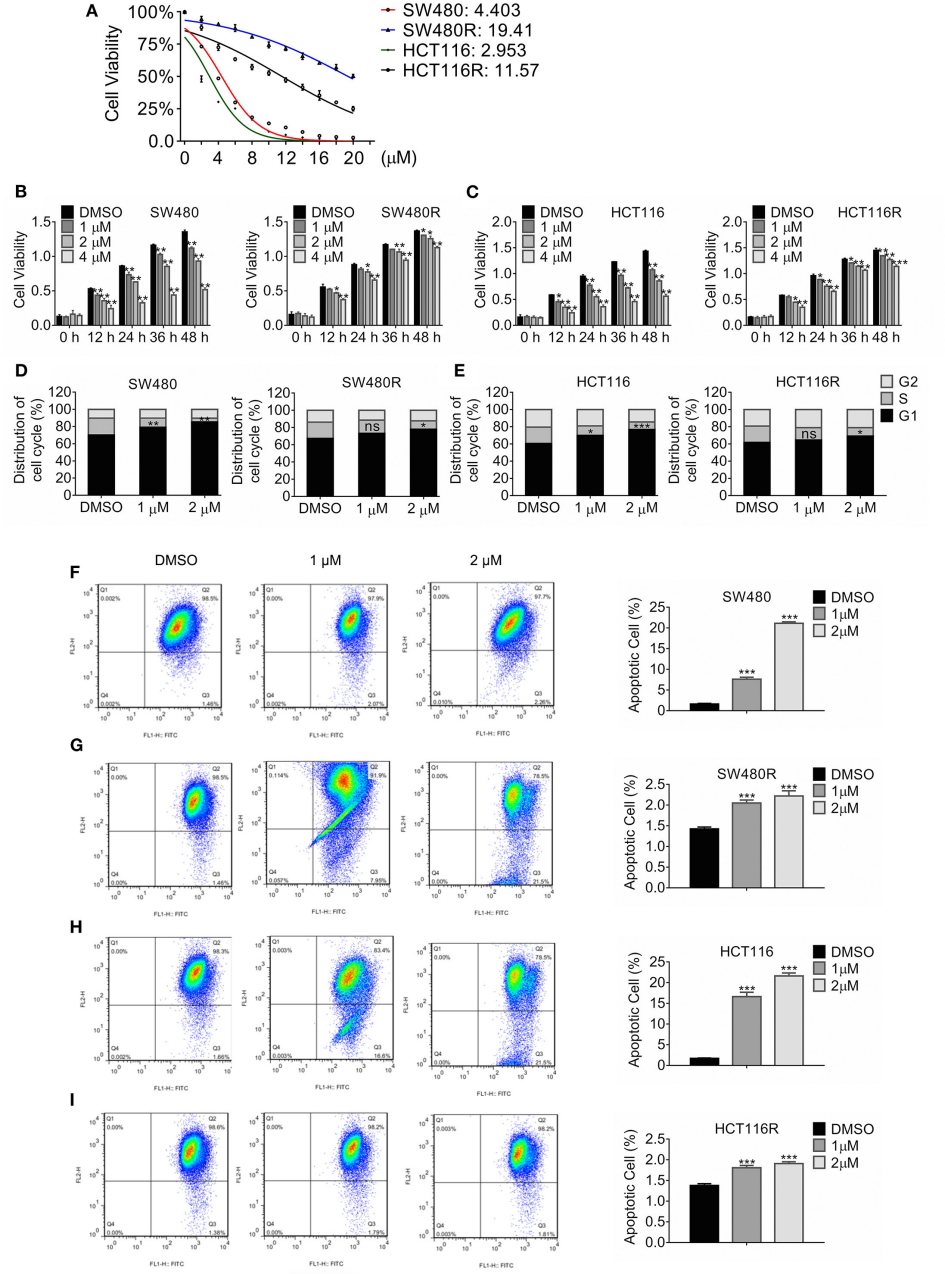
Growth profile of Regorafenib-resistant cells. **(A)** The viability of the indicated cells was detected by MTT assay post-treatment with regorafenib 72 h. The IC50 values for the indicated cells were estimated. Data are mean ± SD (error bars) of three individual experiments. **(B,C)** The cell viability of SW480R and HCT116R, as well as their parental cells, was detected by MTT assay post-treatment with increasing concentrations of regorafenib at the indicated time-point. Data are mean ± SD (error bars) of three individual experiments. *P < 0.05, **P < 0.01, vs. DMSO. **(D,E)** Distribution of cell cycle of SW480R and HCT116R, as well as the parental cells. Cells were exposed to regorafenib or DMSO for 24 h and flow cytometry was performed post DNA staining with propidium iodide. Data obtained from three independent experiments were shown. Data represent mean ± SD. ns, no significance. *P < 0.05, **P < 0.01, ***P < 0.001, vs. DMSO. Apoptosis of SW480 **(F)**, SW480R **(G)**, HCT116 **(H)**, and HCT116R **(I)** were measured by JC-1 apoptosis assay post-exposure of regorafenib or DMSO for 24 h. Data represents mean ± SD of three individual experiments. ***P < 0.001 vs. DMSO.

Colon cancer cell lines SW480 and HCT116 were treated with stepwise escalating regorafenib for over 12 months, and the resultant clones were harvested. We characterized three clones termed SW480R and HCT116R. Figure 1A showed that the adapted subclones developed resistance against regorafenib significantly compared to the parental cells. The half-maximal inhibitory concentration (IC_50_) values for SW480R were approximately five times those for SW480 (19.41 vs. 4.303 μM). Likewise, the IC_50_ values for HCT116R were 11.57 μM, which were four times greater than those for HCT116 (2.953 μM). The IC_50_ values for all subclones of SW480 and HCT116 were shown in Figure S1. The results in Figure 1B indicated that 36 h-treatment with 1 μM regorafenib failed to inhibit the viability of SW480R cells, whereas the same exposure to regorafenib retarded SW480 cells viability significantly. Figure 1C exhibited similar results in HCT116R, where 12 h-exposure to 1 μM regorafenib changed the viability of HCT116R little while inhibited that of HCT116 evidently. Furthermore, Cell cycle distribution of the regorafenib-resistant cells and the parental cells post regorafenib treatment were analyzed by flow cytometry. As shown in Figure 1D, 1 μM regorafenib had little effect on cell cycle distribution of SW480R while that induced significant G1 phase arrest of SW480. Comparably, the HCT116R population in the G1 phase changed hardly, whereas the HCT116 population in the G1 phase increased remarkably post-1 μM regorafenib exposure (Figure 1E). In addition, cell apoptosis was accessed with the JC-1 assay followed by flow cytometry. As shown in Figure 1F, apoptotic SW480 cells raised significantly (1.59 vs. 7.58%) while apoptotic SW480R cells increased slightly (1.42 vs. 2.05%) post-1 μM regorafenib treatment (Figure 1G). Consistently, apoptotic HCT116 cells raised from 1.667 to 16.63% (Figure 1H) while apoptotic HCT116R cells increased from 1.37 to 1.81% post equal regorafenib treatment (Figure 1I). Hence, we performed the following *in vitro* experiments with 1 μM regorafenib. Briefly, these results indicated that we established regorafenib-resistant cells.

### LncRNA MIR570MG Upregulates in Regorafenib-Resistant Cells and Promotes Cell Viability

**Figure 2:**
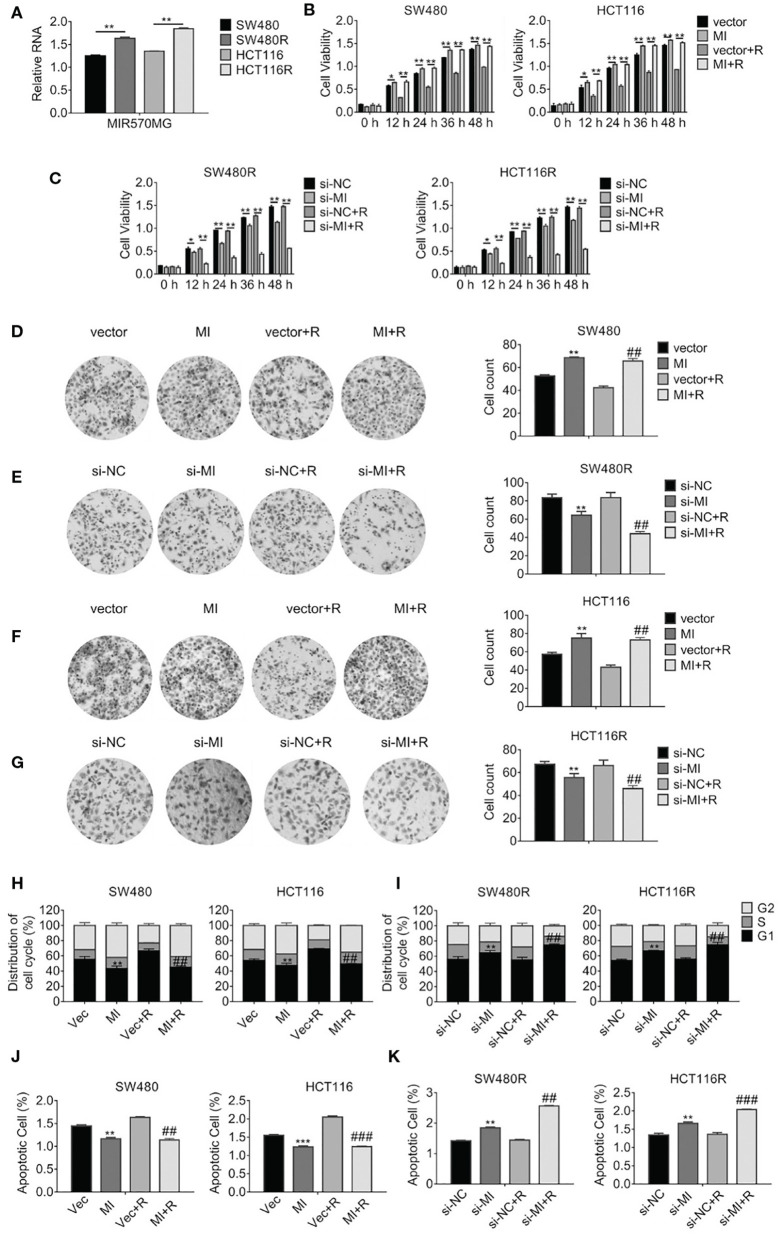
LncRNA MIR570MG expression increases and enhances the cell viability of regorafenib-resistant cell lines. **(A)** Expression of MIR570MG in regorafenib resistant- and parental cells. **(B)** The viability of the indicated cells was accessed by the MTT assay. Cells expressing MIR570MG or vector were exposed to regorafenib. MTT assay was performed at the indicated time-points. **(C)** The viability of the indicated cells was accessed by the MTT assay. Cells expressing si-MIR570MG or si-NC were exposed to regorafenib before MTT assay. **(D–G)** Clonogenicity of the indicated cells was measured by colony formation. Cells were transfected with MIR570MG or si-MIR570MG for 24 h prior to regorafenib treatment and colony formation assay was conducted. **(H,I)** The distribution of the cell cycle of the indicated cells was analyzed by flow cytometry. Cells are expressing MIR570MG or si-MIR570MG at the presence or absence of regorafenib. Twenty-four hours later, the flow cytometry analysis was carried out. **(J,K)** The apoptosis of the indicated cells was accessed by the JC-1 assay, followed by flow cytometry analysis. Cells expressing MIR570MG or si-MIR570MG were exposed to regorafenib. Data represents mean ± SD of three individual experiments. *P < 0.05, **P < 0.01, ***P < 0.001, vs. vector or si-NC; ^*##*^*P* < 0.01, ^*###*^*P* < 0.001, vs. vector or si-NC plus regorafenib exposure. MI, MIR570MG; si-MI, si-MIR570MG; NC, negative control; R, Reg, 1 μM regorafenib exposure.

According to the analysis of the LncRNA microarray, we found the lncRNA MIR570MG increased significantly in regorafenib-resistant cells. The result was validated in cell lines by qRT-PCR analysis. First, MIR570MG distinctively overexpressed in regorafenib-resistant cells in comparison with their corresponding parental cells (Figure 2A). Second, results in Figure 2B revealed that MIR570MG overexpression promoted cell viability and conferred parental cells resistance to regorafenib. Inversely, siRNA targeting MIR570MG impaired cell viability of SW480R and HCT116R and deprived the resistance to regorafenib (Figure 2C). Third, colony formation assay was performed to investigate the effects of MIR570MG on clonogenicity. Results in Figure 2D showed that regorafenib inhibited colony formation of SW480 cells significantly while overexpression of MIR570MG overcame the inhibition. Alternatively, regorafenib had little effect on the clonogenicity of SW480R while knockdown of MIR570MG recovered sensitivity to regorafenib (Figure 2E). Equal results were gain in HCT116 and HCT116R cells, respectively (Figures 2F,G). Furthermore, cell cycle analysis was conducted to explore the effect of regorafenib on cell cycle progress. As seen in Figure 2H, overexpression of MIR570MG in SW480 reduced the G1 phase arrest triggered by regorafenib (vec+R, 66.58 vs. MI+R, 44.68%). Similar results were observed in HCT116 cells. Unlikely, Figure 2I proved that knockdown of MIR570MG in SW480R provoked the G1 phase arrest by regorafenib (si-NC+R, 55.08 vs. si-MI+R, 74.53%), as well as in HCT116R. Additionally, a JC-1 assay was performed to detect the effect of MIR570MG on apoptosis. Figure 2J indicated that ectopic expression of MIR570MG in SW480 reduced apoptosis compared to control (vector+R, 1.634 vs. MI+R, 1.14%), whereas the si-MIR570MG promoted apoptosis post-regorafenib exposure (si-NC+R, 1.441 vs. si-MI+R, 1.14%). Similar results were observed in HCT116 cells (Figure 2K). Conversely, knockdown of MIR570MG triggered apoptosis of regorafenib-resistant cells compared to siRNA negative control and recovered the sensitivity to regorafenib. In brief, we found MIR570MG overexpressed in regorafenib-resistant cells and enhanced cell viability by retarding G1 phase arrest and apoptosis.

### MIR570MG Promotes Cell Viability by Sponging miR-145

**Figure 3:**
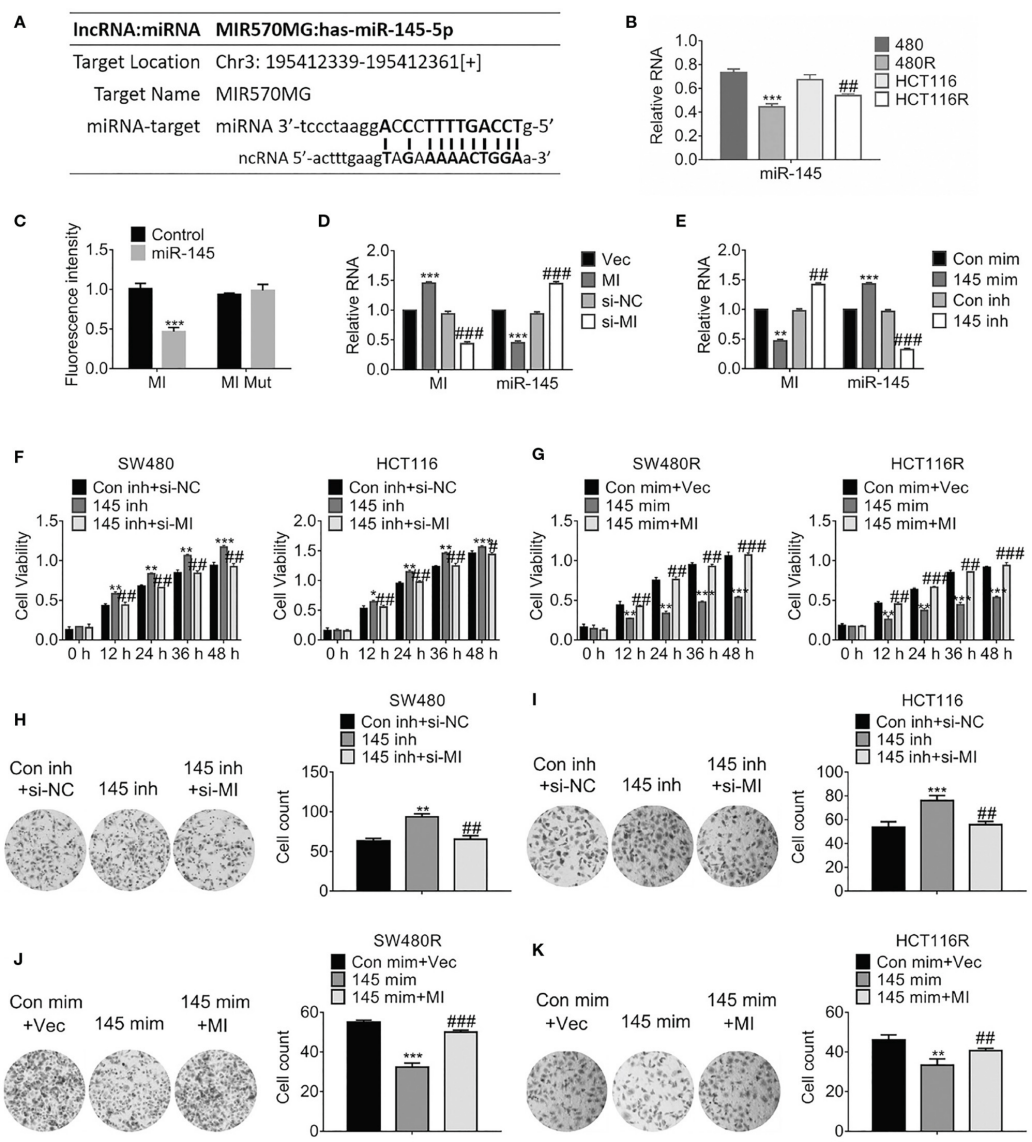
MIR570MG sponges miR-145 and reverses miR-145-mediated growth suppression. **(A)** Schematic diagram of putative binding sites between MIR570MG and miR-145. **(B)** Expression of miR-145 in the indicated cell lines. **P < 0.01, vs. SW480; ^*##*^*P* < 0.01, vs. HCT116. **(C)** Dual-luciferase activity of miR-145 promoter. MI, wild type MIR570MG; MI Mut, MIR570MG mutant. ***P < 0.001, vs. control. **(D)** Expression of the indicated RNA in cells expressing MIR570MG or si-MIR570MG. ***P < 0.001, vs. vector; ^*###*^*P* < 0.001, vs. si-negative control. **(E)** Expression of the indicated RNA in cells expressing miR-145 mimic or miR-145 inhibitor. **P < 0.01, ***P < 0.001, vs. control-mimic; ^*##*^*P* < 0.01, vs. control-inhibitor. **(F)** Viability of SW480 and HCT116 cells was accessed by MTT assay at the indicated time-points. *P < 0.05, **P < 0.01, ***P < 0.001, vs. control; ^#^*P* < 0.05, ^*##*^*P* < 0.01, vs. miR-145 inhibitor. **(G)** Viability of SW480R and HCT116R cells was accessed by MTT assay at the indicated time-points. **P < 0.01, ***P < 0.001, vs. control; ^*##*^*P* < 0.01, ^*###*^*P* < 0.001, vs. miR-145 mimic. **(H,I)** Colony formation of the indicated cells expressing miR-145 inhibitor or miR-145 inhibitor combined with si-MIR570MG. **P < 0.01, vs. control; ^*##*^*P* < 0.01, vs. miR-145 inhibitor. **(J,K)** Colony formation of the indicated cells expressing miR-145 mimic or miR-145 mimic combined with MIR570MG. ***P < 0.001 vs. control; ^*##*^*P* < 0.01 vs. miR-145 mimic. Data represents mean ± SD of three individual experiments. Vec, vector; MI, MIR570MG; si-NC, negative control; si-MI, si-MIR570MG; Con mim, control mimic; 145 mim, miR-145 mimic; Con inh, control inhibitor; R, Reg, 1 μM regorafenib exposure.

Considering the prediction performed by miRbase (http://www.mirbase.org/), we found miR-145 was one of the sponges of MIR570MG, and their potential interaction was shown in Figure 3A. Particularly, miR-145 was deregulated in regorafenib-resistant cells compared to their corresponding parental cells (Figure 3B). To investigate the interaction between MIR570MG and miR-145, we generated the MIR570MG mutant which was deficient in binding miR-145. Dual-luciferase assay was performed with cells expressing MIR570MG or MIR570MG mutant, along with miR-145 mimic. Results in Figure 3C proved that MIR570MG inhibited fluorescence intensity of miR-145 significantly while MIR570MG mutant changed little. The expression of miR-145 decreased notably in cells expressing MIR570MG, whereas that increased in cells expressing si-MIR570MG (Figure 3D). The introduction of miR-145 inhibitor had identical results with ectopic expression of MIR570MG (Figure 3E). To examine the effect of MIR570MG: miR-145 interaction on cell growth, MTT assay was conducted. As shown in Figure 3F, knockdown of miR-145 enhanced the corresponding parental cell growth while the expression of miR-145 inhibitor and si-MIR570MG simultaneously hampered the promotion compared to the control. Consistent with the results, overexpression of miR-145 repressed the growth of regorafenib-resistant cells while co-expression of miR-145 and MIR570MG recovered growth (Figure 3G). Besides, overexpression of miR-145 inhibitor enhanced the colony formation of SW480 and HCT116, whereas the combination of miR-145 inhibitor and si-MIR570MG rescued the clonogenicity (Figures 3H,I). Alternatively, the introduction of miR-145 mimic deregulated the colony formation of SW480R and HCT116R, whereas the combination of miR-145 mimic and MIR570MG provoked the clonogenicity (Figures 3J,K).

### miR-145 Targets SMAD3 and Prohibits SMAD3-Promoted Growth

**Figure 4:**
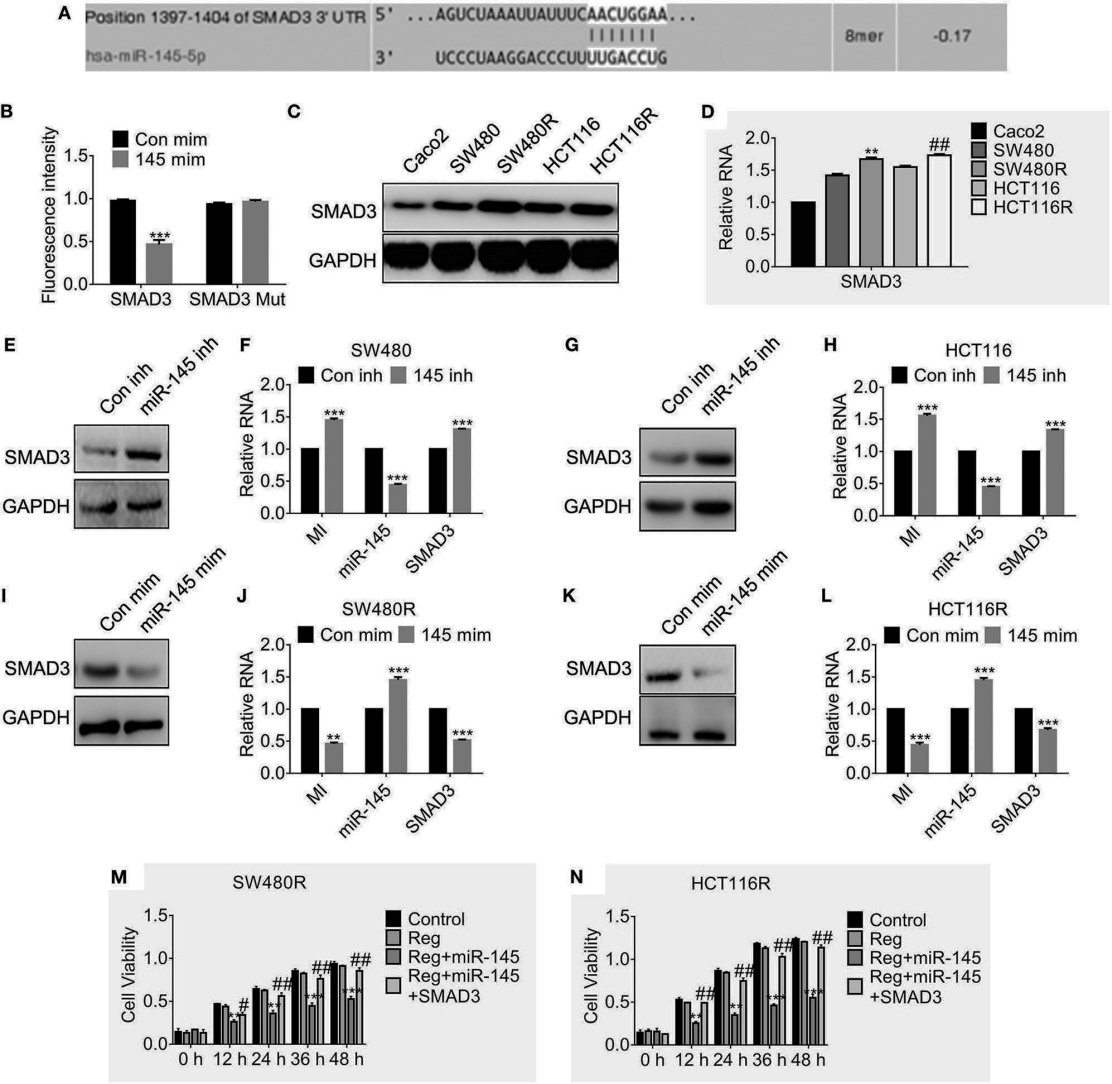
miR-145 targets SMAD3 and deregulated SMAD3-enhanced viability. **(A)** Schematic diagram of predictive binding sites of miR-145 and SMAD3. **(B)** Dual-luciferase activity of SMAD3 promotor. SMAD3, SMAD3 wild type 3′-UTR; SMAD3 MUT, SMAD3 mutant 3′-UTR. **(C,D)** The expression of SMAD3 in the indicated cells were accessed by western blot and qRT-PCR, separately. **P < 0.05, vs. SW480. ^*##*^*P* < 0.05, vs. HCT116. **(E–H)** The expression of SMAD3, MIR570MG and miR-145 in the indicated cells expressing miR-145 inhibitor. inh, inhibitor. ***P < 0.001, vs. control. **(I–L)** The expression of SMAD3, MIR570MG and miR-145 in the indicated cells expressing miR-145 mimic. mim, mimic. ***P < 0.001, vs. control. **(M,N)** The cell viability of the indicated cells was accessed by the MTT assay. Cells expressing miR-145 or miR-145 plus SMAD3 were exposed to 1 μM regorafenib. MTT assay was performed post-treatment at the indicated time-point. **P < 0.01, ***P < 0.001, vs. regorafenib; ^*##*^*P* < 0.01, vs. miR-145 exposed to Regorafenib. Data obtained from independent experiments thrice. Con mim, control mimic; 145 mim, miR-145 mimic; Con inh, control inhibitor; Reg, 1 μM regorafenib exposure.

In light of the prediction of miRDB, we found miR-145 targeted SMAD3 and the estimated binding sites were shown in Figure 4A. To investigate the details, we constructed the SMAD3 mutant where the 3′-untranslational regions of SMAD3 failed to combine miR-145. Figure 4B suggested the fluorescence intensity of SMAD3 fell significantly while that of the SMAD3 mutant was constant compared to the control. Importantly, SMAD3 was upregulated in the regorafenib-resistant cells compared to their parental cells on protein levels (Figure 4C) and mRNA levels (Figure 4D). Furthermore, forced expression of miR-145 inhibitor promoted the SMAD3 expression (Figures 4E–H). Conversely, expression of miR-145 mimic attenuated the SMAD3 expression in SW480 (Figures 4I–L). To access the effect of miR-145 on viability, cells were transfected with miR-145 or combination of miR-145 and SMAD3 before regorafenib exposure. As shown in Figures 4M,N, miR-145 overexpression rehabilitated the response to regorafenib; however, simultaneous expression of SMAD3 and miR-145 recovered the resistance against regorafenib in SW480R and HCT116R cells.

### MIR570MG Confers Resistance Against Regorafenib via Rescuing SMAD3 From miR-145

**Figure 5:**
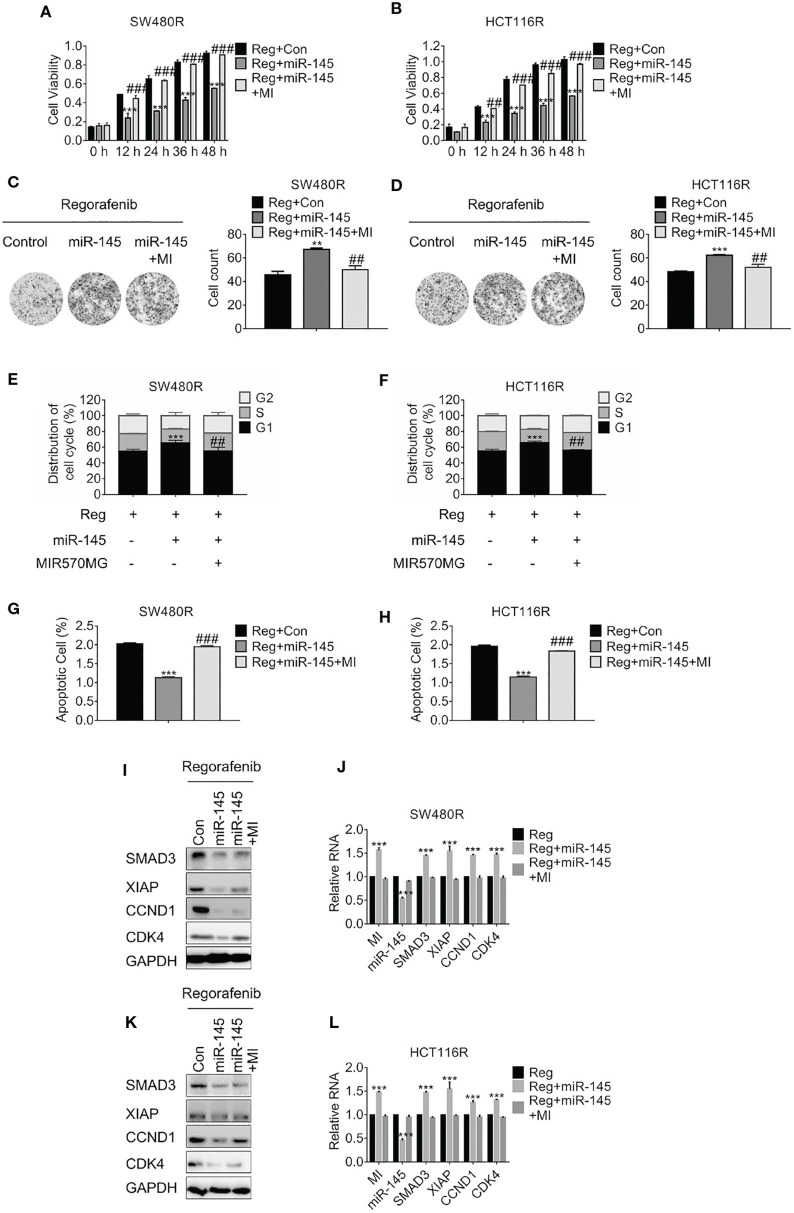
MIR570MG confers resistance against regorafenib by releasing SMAD3 from miR-145. **(A,B)** Cell viability of SW480R and HCT116R was accessed by MTT assay. Cells expressing miR-145 or miR-145 plus MIR570MG were treated with Regorafenib. MTT assay was conducted post-treatment at different time-points. **(C,D)** Colony formation of SW480R and HCT116R was performed post-regorafenib treatment. Cells were transfected with MIR570MG or the combination prior to regorafenib treatment. **(E,F)** Distribution of cell cycle of the indicated cells were analyzed by flow cytometry. Cells expressing miR-145 or the combination were exposed to 1 μM regorafenib for 24 h prior to flow cytometry analysis. **(G,H)** Apoptosis of SW480R and HCT116R was measured post-regorafenib treatment. Cells expressing miR-145 or combination were exposed to 1 μM regorafenib for 24 h. JC-1 assay was performed, followed by flow cytometry analysis post-treatment. The expression of the indicated genes in SW480R **(I,J)** and HCT116R **(K,L)** cells were measured by Western blot and qRT-PCR, separately. **P < 0.01, ***P < 0.001, vs. control exposed to regorafenib; ^*##*^*P* < 0.01, ^*###*^*P* < 0.001, vs. miR-145 exposed to regorafenib. Data obtained from three independent experiments. MI, MIR570MG; Reg, 1 μM regorafenib exposure.

To investigate the effect of MIR570MG: miR-145 interaction on cell viability, MTT assay was carried out. The results in Figures 5A,B showed the miR-145 diminished SW480R and HCT116R cell growth apparently, while MIR570MG recovered the growth. Moreover, the amounts of SW480R clones expressing miR-145 alone dropped obviously, whereas those expressing combination equaled control (Figure 5C). The identical results were observed in HCT116R cells (Figure 5D). Furthermore, overexpression of miR-145 in SW480R caused prominent G1 phase arrest while expression of miR-145 and MIR570MG concurrently induced equal G1 phase arrest compared to control (Figure 5E), as well as in HCT116R (Figure 5F). Meanwhile, Figures 5G,H proved that miR-145 promoted apoptosis of regorafenib-resistant cells, while the combination triggered similar apoptosis in comparison with control. To clarify the MIR570MG: miR-145 mediated mechanisms, SMAD3, apoptosis suppressor XIAP, G1/S transition key regulate complex Cyclin D1: Cyclin-dependent kinase 4 were determined. The expression of SMAD3, Cyclin D1, and CDK4 dropped in cells expressing miR-145 while XIAP raised (Figures 5I,K). The alteration of the mRNA agreed with that of protein (Figures 5J,L). To sum up, MIR570MG sponged miR-145 and retrieved SMAD3, activating SMAD3-related signaling cascades. As a result, cells acquired resistance to regorafenib.

### MIR570MG Restores Resistance Against Regorafenib by Sponging miR-145 in HCT116R Mouse Model

**Figure 6:**
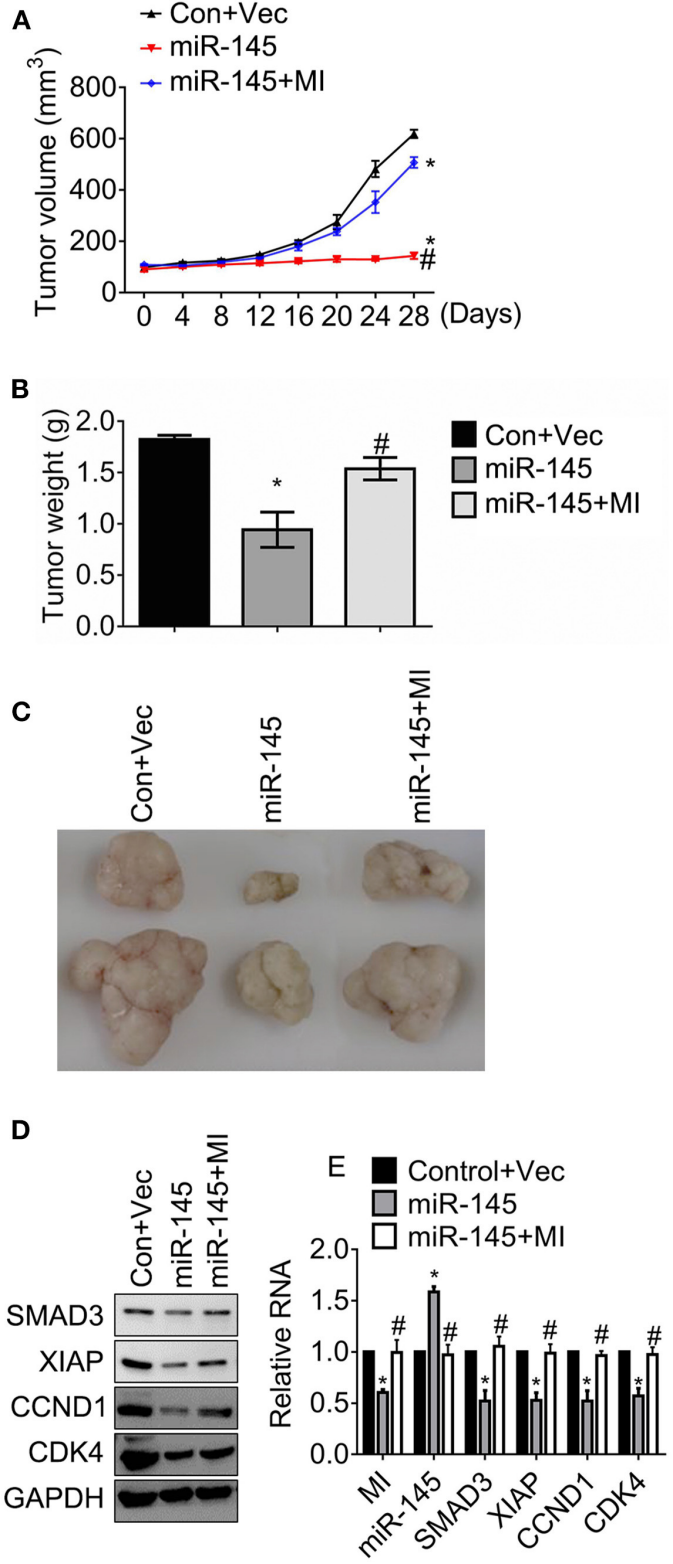
MIR570MG restores resistance against regorafenib by sponging miR-145 in HCT116R xenografts. Mice carrying ~100mm^3^ tumors were divided into three groups randomly (six per group) and tumor volumes were measured. **(A)** Tumor growth curves of HCT116R mouse models. **(B)** Tumor weights of HCT116R mouse models. **(C)** Representative images of tumors in each group. **(D)** The indicated protein expression was accessed by immunoblot. **(E)** The indicated lncRNA, miRNA and mRNA expression were analyzed by qRT-PCR. Data obtained from three independent experiments. *P < 0.05, vs. control. ^#^*P* < 0.05, vs. miR-145. Con, control; Vec, vector; MI, MIR570MG.

The effects of MIR570MG on tumor growth was determined in an HCT116R mouse model. After the establishment of tumors, mice received daily 30 mg/kg regorafenib or the corresponding vehicle for 4 weeks. The tumor volumes were recorded every 4 days and the tumors were weighed at day 28 as the indicator of tumor burden. The volumes of tumors carrying miR-145 kept stable for 28 days while those of control developed fast (Figure 6A). For comparison, the tumors expressing miR-145 and MIR570MG overgrew, suggesting that MIR570MG recovered the resistance to regorafenib by overcoming miR-145. In line with the alteration of tumor growth curves, the mean tumor weights of miR-145 alone dropped significantly (0.9 ± 0.2 vs. 1.8 ± 0.1 g), whereas those of combination fell moderately (1.5 ± 0.1 vs. 1.8 ± 0.1 g) in comparison with control (Figure 6B). Figure 6C showed the representative images of tumors that were consistent with the volume measurement in each group. Moreover, the expression of Smad3, XIAP, Cyclin D1, and Cdk4 reduced notably in tumors expressing miR-145 while that increased in tumors expressing the combination (Figure 6D), indicating that the changes *in vivo* were coinciding with those *in vitro*. Additionally, the stable transfection efficiency of MIR570MG and miR-145 was confirmed by qRT-PCR (Figure 6E). The alterations of the Smad3-regulated genes on RNA levels were identical to the changes in protein levels.

## Discussion

Regorafenib is an oral multi-kinases inhibitor that targets angiogenesis-related genes, including VEGFR1, VEGFR2, VEGFR3, and TIE2, as well as various oncogenes such as KIT, RET, RAF1, BRAF, PDGFR, FGFR, and p38 MAPK (11). Regorafenib has been used for late line treatment for several cancers including advanced colorectal cancer (12), hepatocellular carcinoma (13), and gastrointestinal stromal tumors (14). Particularly, regorafenib monotherapy for treating metastatic colorectal cancer was approved by the U.S. Food and Drug Administration due to the 1.5 months improvement in the CORRECT trial compared to the placebo (15). Despite the favorable outcome of regorafenib regimens in metastatic colorectal cancer, the primary and adaptive resistance to regorafenib restrains application in refractory and recurrent colorectal cancer. Given the characteristic of the multi-kinase suppression, potential mechanisms of resistance against regorafenib vary. Kort et al. demonstrated the multidrug efflux transporters Breast Cancer Resistance Protein and P-Glycoprotein hampered brain and testis accumulation of regorafenib in mice model, leading to limited response of brain metastasis (16). Ohya et al. indicated multidrug resistance protein 2 and organic anion transporting polypeptide 1B1 transported regorafenib (17). Mirone et al. evaluated the upregulated Notch-1 drove resistance to regorafenib in colorectal cancer cells (18). Tutusaos et al. elucidated the antiapoptotic BCL-2 proteins determined regorafenib resistance in hepatocellular carcinoma (19). Furthermore, Wang et al. revealed the unique isomerase Pin1 triggered the acquired resistance to regorafenib via activating epithelial-mesenchymal transition in hepatocellular carcinoma cells (20). Recently, Ou et al. proved the CC chemokine receptor 2 and β-catenin were involved in a positive-feedback loop and conferred tolerance to regorafenib in colorectal cancer (21). To date, compelling evidence has shown lncRNAs were involved in cancer progression by targeting different signaling pathways, including induction of chemo-, radio-, and targeted therapy resistance. However, the association of lncRNAs and resistance to regorafenib remains unknown. The present results that lncRNA MIR570MG was upregulated in acquired regorafenib-resistant cells filled the gaps among lncRNAs and the resistance against regorafenib. Overexpression of MIR570MG promoted cell survival, colony formation while reduced the G1 phase arrest, leading to antagonism toward regorafenib exposure.

Increasing evidence indicates that lncRNAs regulate gene expression post-transcriptionally by interacting with miRNAs. Like a sponge, lncRNAs bind to miRNAs with complementary sequences. For instance, lncRNA CRNDE enhanced chemoresistance via miR-181a-5p-mediated Wnt/β-catenin cascades (22). lncRNA MIR100HG-derived miR-100, together with miR-125b, promoted cetuximab resistance by activation of Wnt/β-catenin signaling (23). lncRNA KCNQ1OT1 inhibited miR-760/PPP1R1B and enhanced the methotrexate resistance via the cAMP signaling pathway (24). Our study validated that miR-145 as one of the sponges of MIR570MG, deregulated in the parental colon cancer cells while upregulated in the regorafenib-resistant cells. Furthermore, overexpression of miR-145 mimic, as well as a diminishment of MIR570MG, allowed the regorafenib-resistant cells regained response to regorafenib. Further investigation revealed that miR-145 bound to the 3′-UTR of *SMAD3* directly and reduced Smad3 expression. Apart from competing interacting with *SMAD3* in acquired regorafenib-resistance, miR-145 antagonized multiple oncogenes in colorectal cancer progression. For example, miR-145 confronted Snai1 and attenuated Snai1-mediated stemness and radiation resistance in colorectal cancer (25). Transcriptional modulator TCF4/β-catenin complex and histone trimethylation complex PRC2 repressed miR-145 simultaneously, initiated the negative feedback loops and promoted tumor progression (26). To describe the divergent roles of miR-145 in cancer progression, a profound understanding and global picture of miR-145 across multiple cancers would be essential.

Activation of Smad3 is pivotal in the TGFβ/Smad pathway via the interaction of TGFβ and receptor. Phosphor-TGFβ receptor phosphorylates and initiates Smad2 and Smad3; therefore, the trimeric complex Smad2: Smad3: Smad4 establishes and triggered cell growth and survival (27). In the present study, we showed miR-145 reduced *SMAD3* expression *in vitro* and *in vivo*, along with the decrease of Cdk4/Cyclin D1 and XIAP. The results were consistent with the previous report that Cdk4 inhibition dephosphorylated Smads (28). The results suggested that miR-145 worked as a tumor suppressor in acquired regorafenib-resistant colon cancer cells.

Although the above findings extend the knowledge of MIR570MG-mediated resistance to regorafenib, further experiments to elucidate the interaction between tumor cells and microenvironment may be required. Furthermore, to explore the expression of MIR570MG in different stages of colon cancer, clinical specimens of pre- or post-targeted therapy, as well as the recurrent tumor samples, should be collected. Besides, the alteration of genomics, at least the regorafenib-targeted oncogenes, should be clarified.

In summary, we provided evidence that lncRNA MIR570MG augmented in regorafenib-resistant colon cancer cells and conferred resistance by sponging with miR-145, leading to the promotion of Smad3-mediated cell survival, colony formation, and tumor growth.

## Data Availability Statement

The datasets generated for this study can be found in the NCBI Gene Expression Omnibus (GSE141134).

## Ethics Statement

The animal study was reviewed and approved by the Medical Ethics Committee of the Fourth Affiliated Hospital of China Medical University.

## Author Contributions

FW wrote the main manuscript. MW, ZL, and YW performed the experiments. FW and YZ designed the research. FW, MW, and YZ performed data analysis. ZL, YW, and YZ contributed to manuscript revisions. All authors reviewed the manuscript and read and approved the final manuscript.

### Conflict of Interest

The authors declare that the research was conducted in the absence of any commercial or financial relationships that could be construed as a potential conflict of interest.

## Supplementary Material

Figure S1 The IC_50_ values for three subclones of SW480 and HCT116.
