# Corrigendum: Long Non-Coding RNA MIR570MG Causes Regorafenib Resistance in Colon Cancer by Repressing miR-145/SMAD3 Signaling

**DOI:** 10.3389/fonc.2021.818876

**Published:** 2022-01-07

**Authors:** Fang Wei, Mofei Wang, Zhen Li, Yong Wang, Yong Zhou

**Affiliations:** Department of General Surgery, The Fourth Affiliated Hospital of China Medical University, Shenyang, China

**Keywords:** LncRNA MIR570MG, miR-145, SMAD3, TGFβ, metastatic colon cancer, regorafenib, resistance

In the original article, there were mistakes in **Figures 2**, **3**, and **5** as published. Parts of images in the above-mentioned figures overlapped. We inspected the original data and found that parts of raw images were placed in a single fold without labeling them correctly. The authors selected the images in which cell clusters were clear and the numbers could be precisely counted. During the selection, some images were inappropriately presented. The corrected **Figures 2**, **3**, and **5** appear below.

**Figure 2:**
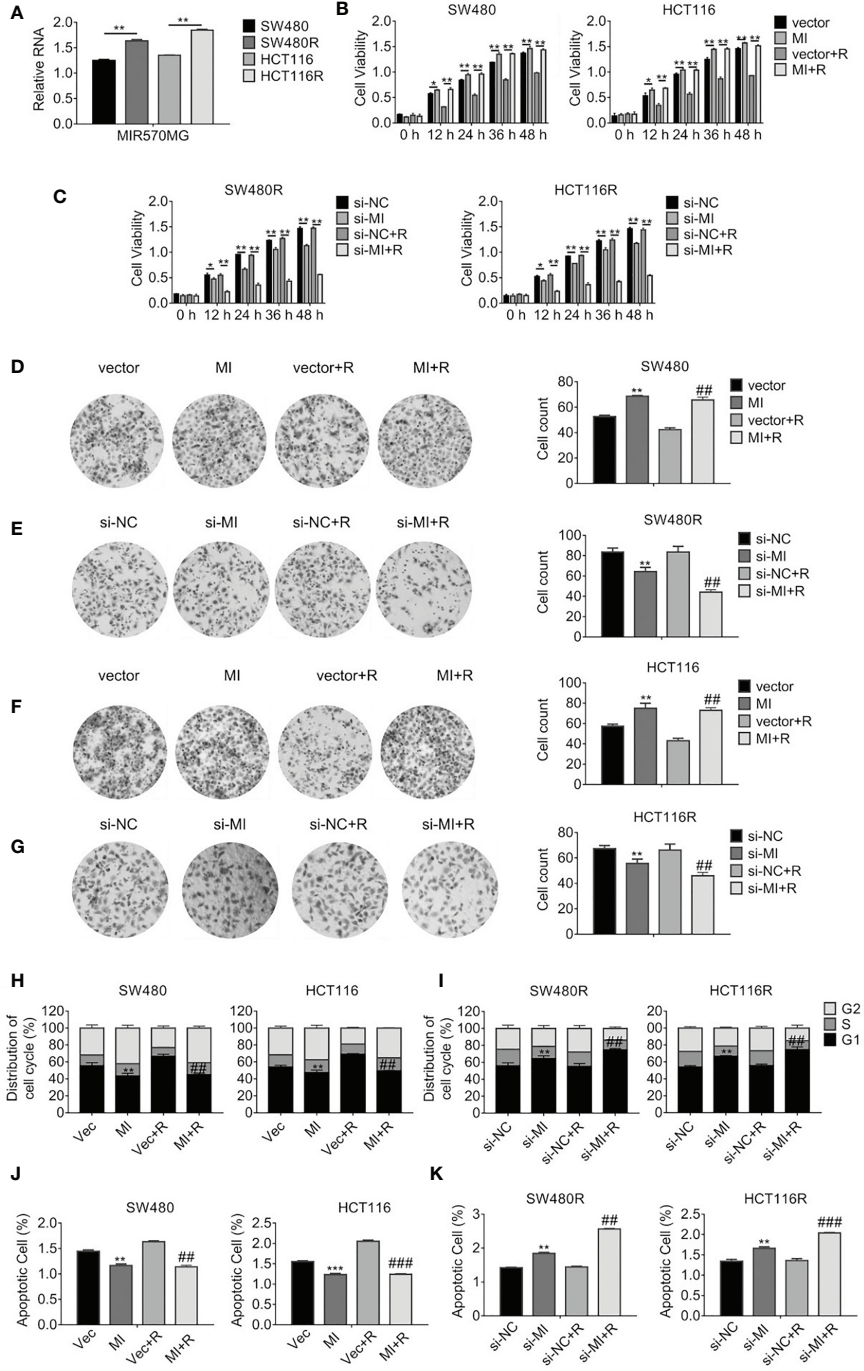
LncRNA MIR570MG expression increases and enhances the cell viability of regorafenib-resistant cell lines. **(A)** Expression of MIR570MG in regorafenib resistant- and parental cells. **(B)** The viability of the indicated cells was accessed by the MTT assay. Cells expressing MIR570MG or vector were exposed to regorafenib. MTT assay was performed at the indicated time-points. **(C)** The viability of the indicated cells was accessed by the MTT assay. Cells expressing si-MIR570MG or si-NC were exposed to regorafenib before MTT assay. **(D–G)** Clonogenicity of the indicated cells was measured by colony formation. Cells were transfected with MIR570MG or si-MIR570MG for 24 h prior to regorafenib treatment and colony formation assay was conducted. **(H, I)** The distribution of the cell cycle of the indicated cells was analyzed by flow cytometry. Cells are expressing MIR570MG or si-MIR570MG at the presence or absence of regorafenib. Twenty-four hours later, the flow cytometry analysis was carried out. **(J, K)** The apoptosis of the indicated cells was accessed by the JC-1 assay, followed by flow cytometry analysis. Cells expressing MIR570MG or si-MIR570MG were exposed to regorafenib. Data represents mean ± SD of three individual experiments. *P < 0.05, **P < 0.01, ***P < 0.001, vs. vector or si-NC; ^##^P < 0.01, ^###^P < 0.001, vs. vector or si-NC plus regorafenib exposure. MI, MIR570MG; si-MI, si-MIR570MG; NC, negative control; R, Reg, 1 μM regorafenib exposure.

**Figure 3:**
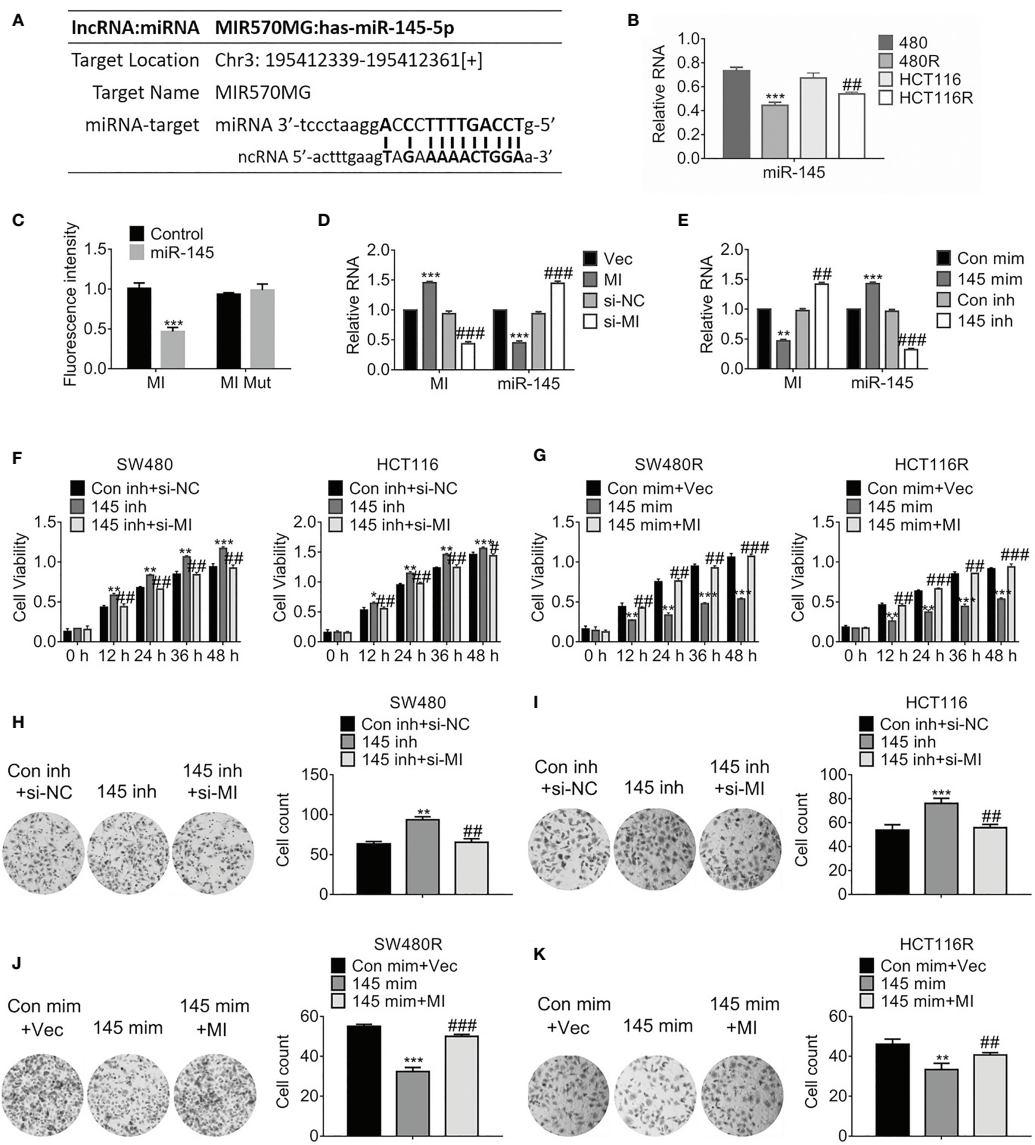
MIR570MG sponges miR-145 and reverses miR-145-mediated growth suppression. **(A)** Schematic diagram of putative binding sites between MIR570MG and miR-145. **(B)** Expression of miR-145 in the indicated cell lines. **P < 0.01, vs. SW480; ^##^P < 0.01, vs. HCT116. **(C)** Dual-luciferase activity of miR-145 promoter. MI, wild type MIR570MG; MI Mut, MIR570MG mutant. ***P < 0.001, vs. control. **(D)** Expression of the indicated RNA in cells expressing MIR570MG or si-MIR570MG. ***P < 0.001, vs. vector; ^###^P < 0.001, vs. si-negative control. **(E)** Expression of the indicated RNA in cells expressing miR-145 mimic or miR-145 inhibitor. **P < 0.01, ***P < 0.001, vs. control-mimic; ^##^P < 0.01, vs. control-inhibitor. **(F)** Viability of SW480 and HCT116 cells was accessed by MTT assay at the indicated time-points. *P < 0.05, **P < 0.01, ***P < 0.001, vs. control; ^#^P < 0.05, ^##^P < 0.01, vs. miR-145 inhibitor. **(G)** Viability of SW480R and HCT116R cells was accessed by MTT assay at the indicated time-points. **P < 0.01, ***P < 0.001, vs. control; ^##^P < 0.01, ^###^P < 0.001, vs. miR-145 mimic. **(H, I)** Colony formation of the indicated cells expressing miR-145 inhibitor or miR-145 inhibitor combined with si-MIR570MG. **P < 0.01, vs. control; ^##^P < 0.01, vs. miR-145 inhibitor. **(J, K)** Colony formation of the indicated cells expressing miR-145 mimic or miR-145 mimic combined with MIR570MG. ***P < 0.001 vs. control; ^##^P < 0.01 vs. miR-145 mimic. Data represents mean ± SD of three individual experiments. Vec, vector; MI, MIR570MG; si-NC, negative control; si-MI, si-MIR570MG; Con mim, control mimic; 145 mim, miR-145 mimic; Con inh, control inhibitor; R, Reg, 1 μM regorafenib exposure.

**Figure 5:**
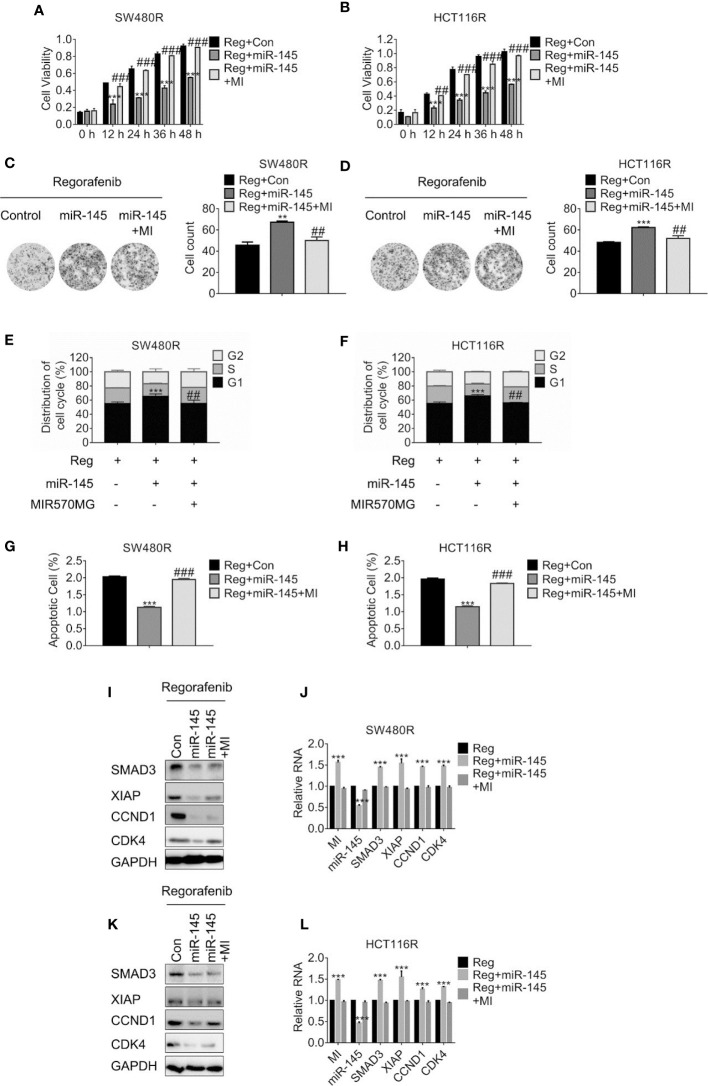
MIR570MG confers resistance against regorafenib by releasing SMAD3 from miR-145. **(A, B)** Cell viability of SW480R and HCT116R was accessed by MTT assay. Cells expressing miR-145 or miR-145 plus MIR570MG were treated with Regorafenib. MTT assay was conducted post-treatment at different time-points. **(C, D)** Colony formation of SW480R and HCT116R was performed post-regorafenib treatment. Cells were transfected with MIR570MG or the combination prior to regorafenib treatment. **(E, F)** Distribution of cell cycle of the indicated cells were analyzed by flow cytometry. Cells expressing miR-145 or the combination were exposed to 1 μM regorafenib for 24 h prior to flow cytometry analysis. **(G, H)** Apoptosis of SW480R and HCT116R was measured post-regorafenib treatment. Cells expressing miR-145 or combination were exposed to 1 μM regorafenib for 24 h. JC-1 assay was performed, followed by flow cytometry analysis post-treatment. The expression of the indicated genes in SW480R **(I, J)** and HCT116R **(K, L)** cells were measured by Western blot and qRT-PCR, separately. **P < 0.01, ***P < 0.001, vs. control exposed to regorafenib; ^##^P < 0.01, ^###^P < 0.001, vs. miR-145 exposed to regorafenib. Data obtained from three independent experiments. MI, MIR570MG; Reg, 1 μM regorafenib exposure.

The authors apologize for this error and state that this does not change the scientific conclusions of the article in any way. The original article has been updated.

## Publisher’s Note

All claims expressed in this article are solely those of the authors and do not necessarily represent those of their affiliated organizations, or those of the publisher, the editors and the reviewers. Any product that may be evaluated in this article, or claim that may be made by its manufacturer, is not guaranteed or endorsed by the publisher.

