# Supplementary figures and images for: Long Non-coding RNA MIR570MG Causes Regorafenib Resistance in Colon Cancer by Repressing miR-145/SMAD3 Signaling
Source: Front Oncol. 2020 Mar 5;10:291. doi: 10.3389/fonc.2020.00291 (PMC7066208; doi:10.3389/fonc.2020.00291)

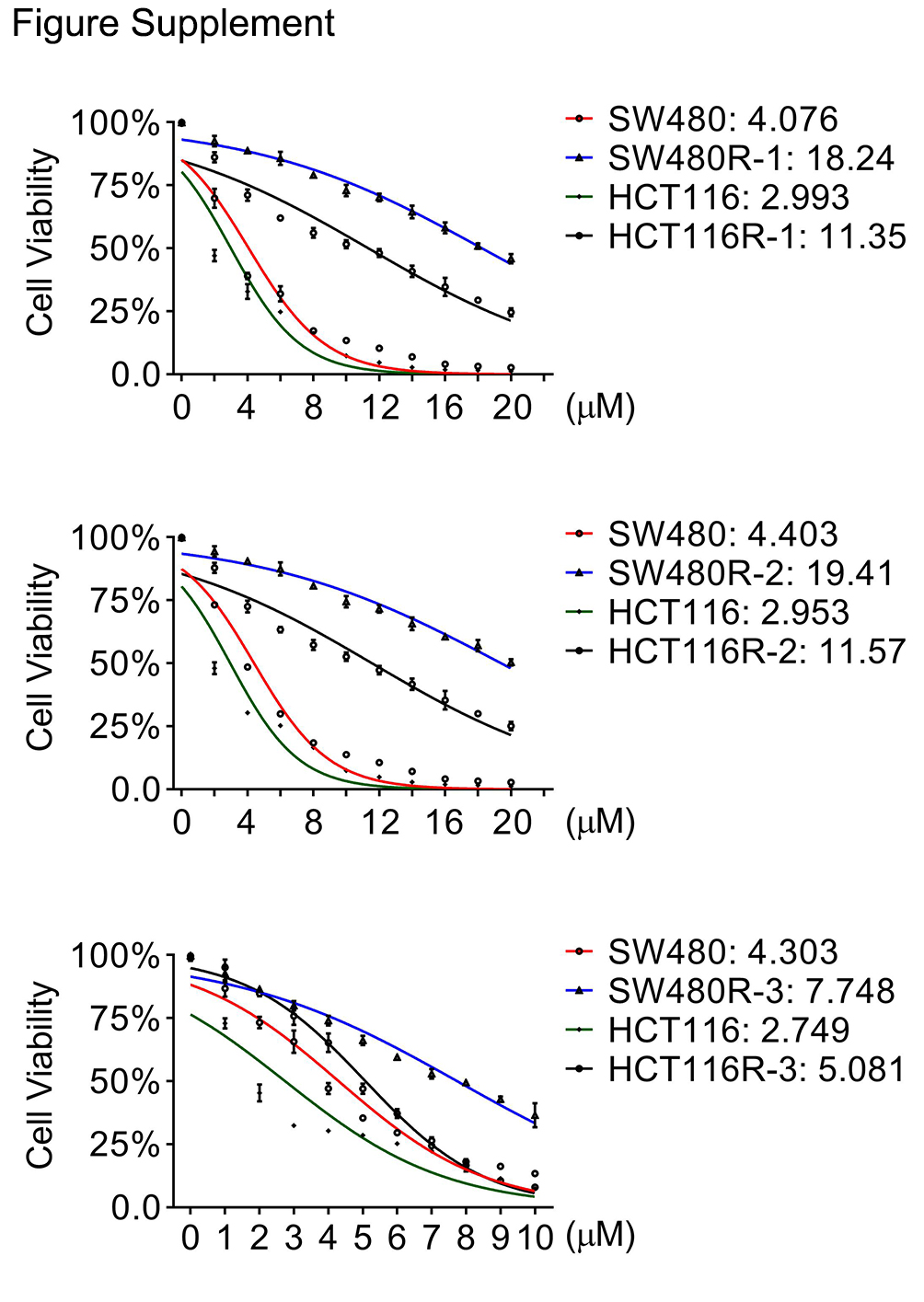

Supplement: Figure S1 — The IC50 values for three subclones of SW480 and HCT116. [file Image_1.jpeg]
